# Associative and Physical Mapping of Markers Related to *Fusarium* in Maize Resistance, Obtained by Next-Generation Sequencing (NGS)

**DOI:** 10.3390/ijms23116105

**Published:** 2022-05-29

**Authors:** Aleksandra Sobiech, Agnieszka Tomkowiak, Bartosz Nowak, Jan Bocianowski, Łukasz Wolko, Julia Spychała

**Affiliations:** 1 Department of Genetics and Plant Breeding, Poznań University of Life Sciences, Dojazd 11, 60-632 Poznań, Poland; agnieszka.tomkowiak@up.poznan.pl (A.T.); julia.spychala@up.poznan.pl (J.S.); 2 Smolice Plant Breeding Ltd., Co., National Research Institute Group, Smolice 146, 63-740 Kobylin, Poland; nowak@hrsmolice.pl; 3 Department of Mathematical and Statistical Methods, Poznań University of Life Sciences, Wojska Polskiego 28, 60-637 Poznań, Poland; jan.bocianowski@up.poznan.pl; 4 Department of Biochemistry and Biotechnology, Poznań University of Life Sciences, Dojazd 11, 60-632 Poznań, Poland; lukasz.wolko@up.poznan.pl

**Keywords:** maize, fusarium, molecular markers, NGS, association mapping

## Abstract

On the basis of studies carried out in the last few years, it is estimated that maize diseases cause yield losses of up to 30% each year. The most dangerous diseases are currently considered to be caused by fungi of the genus *Fusarium*, which are the main culprits of root rot, ear rots, and stalk rot. Early plant infection causes grain diminution, as well as a significant deterioration in nutritional value and fodder quality due to the presence of harmful mycotoxins. Therefore, the aim of the research was to identify new markers of the SilicoDArT and SNP type, which could be used for the mass selection of varieties resistant to fusarium. The plant material consisted of 186 inbred maize lines. The lines came from experimental plots belonging to two Polish breeding companies: Plant Breeding Smolice Ltd., (Co., Kobylin, Poland). Plant Breeding and Acclimatization Institute—National Research Institute Group (51°41′23.16″ N, 17°4′18.241″ E), and Małopolska Plant Breeding Kobierzyce, Poland Ltd., (Co., Kobierzyce, Poland) (50°58′19.411″ N, 16°55′47.323″ E). As a result of next-generation sequencing, a total of 81,602 molecular markers were obtained, of which, as a result of the associative mapping, 2962 (321 SilicoDArT and 2641 SNP) significantly related to plant resistance to fusarium were selected. Out of 2962 markers significantly related to plant resistance in the fusarium, seven markers (SilicoDArT, SNP) were selected, which were significant at the level of 0.001. They were used for physical mapping. As a result of the analysis, it was found that two out of seven selected markers (15,097—SilicoDArT and 58,771—SNP) are located inside genes, on chromosomes 2 and 3, respectively. Marker 15,097 is anchored to the gene encoding putrescine N-hydroxycinnamoyltransferase while marker 58,771 is anchored to the gene encoding the peroxidase precursor 72. Based on the literature data, both of these genes may be associated with plant resistance to fusarium. Therefore, the markers 15,097 (SilicoDArT) and 58,771 (SNP) can be used in breeding programs to select lines resistant to fusarium.

## 1. Introduction

Maize (*Zea mays* L.), along with rice, is the most commonly cultivated crop for, inter alia, human and animal consumption [1]. In the last decade, many factors such as the increase in air temperatures, expansion of the acreage and intensification of cultivation, the introduction of agrotechnical simplifications, and the emergence of new species pathogens have significantly increased the threat to the height and quality of maize crops. It is estimated that maize diseases cause yield losses of up to 30% each year. The quality of the yield also deteriorates [2].

At present, the most dangerous diseases caused by fungi of the genus *Fusarium* spp., which are the main culprits of root rot, ear rots stalk rot., Ear rot, except in cases of severe occurrence, causes slight losses in yield, but greatly deteriorates the quality of grain and fodder as a product for further processing [3].

Often, the symptoms of fusariosis caused by fungi of the genus *Fusarium* spp. are not clearly visible on the cob, but inside, the infestation progresses, leading to the accumulation of mycotoxins [4]. Secondary metabolites of these pathogens contained in food and feed obtained from contaminated grain are very harmful to humans and animals [5,6]. The most common culprit of ear rot is the fungi *Fusarium graminearum* (producing deoxynivalenol—DON and zearalenone—ZON) and *Fusarium verticillioides* (producing fumonisins—FUM).

In addition to the above-mentioned, they can also be accumulated in caryopsis and other parts: trichothecenes, among others T-2 toxin and diacetoksyscirpenol-DAS, ochratoxin A, HT-2 toxin, alphatoxins, etc. These substances can cause many diseases in humans, including various types of allergies, hormonal disorders, and cancer (they activate oncogenic cells). Their presence in the feed is also a great threat to the health and life of animals, especially pigs and poultry, because they cause increased sensitivity to infectious agents that under standard conditions, without the additional action of toxin-producing fungal metabolites, would not be able to cause disease. In addition, they negatively affect production results [7].

In 2007, the European Union introduced standards defining the maximum content of mycotoxins in maize grain (EC No. 1126/2007). If the content of DON in unprocessed grain exceeds 1700 µg/kg, ZEA 350 µg/kg, and FUM 4000 µg/kg, such grain is not eligible for feed use. Breeding and using in the cultivation of varieties less susceptible to infection by fungi of the genus *Fusarium* ssp. Are widely recognized as the most cost-effective and environmentally friendly method of protecting plants against disease infestation [3,8]. In the case of maize, the use of fungicides is difficult and often ineffective, because it is difficult to assess the severity of the disease [9]. Fusarium infestation of plants can also be minimized by reducing the occurrence of pests that damage the corn cobs during feeding [10,11].

Fusarium resistance is a polygenic trait and is strongly influenced by environmental factors. This type of resistance is very complex, making it difficult to breed, and with the result that most commercial maize hybrids have a lower level of resistance than desired [12].

In the era of rapid development of molecular biology tools, it is important to identify markers related to genes influencing resistance to fusarium—in order to facilitate the selection of resistant genotypes. Maschietto et al. [13] demonstrated the usefulness of SSR, GBS markers, transcriptomics, and QLT mapping to improve the selection of lines resistant to fusarium.

In breeding programs, it is recommended to use genomic selection to use molecular markers importantly related to immunity as permanent effects in genotypic value prediction models [14].

Rapid advances in Next Generation Sequencing (NGS) have made it possible to sequence the genome of many crops. High-throughput genotyping methods such as GBS and SNP enable rapid genome profiling to provide growers with detailed information on traits relevant to cultivation. High-resolution genotyping may therefore be the key to revitalizing phenotypic diversity in response to climate change [15].

Next-generation sequencing (NGS) and microarray methods have been used to identify the molecular mechanisms involved in *F. verticillioides* infection in resistant and susceptible maize genotypes [16,17]. All of these studies compared the response of resistant and susceptible lines to infection taking into account the early (12–48 h post-inoculation) and late (72–120 h post-inoculation) stages of infection. RNAseq made it possible to identify several thousand genes with different expressions and led to the possibility of discovering new genes expressed [18].

Diversity Arrays Technology (DArT) is a technology that does not require sequence information [19]. It uses a number of clones resulting from the amplification of restriction fragments. This method allows the screening of hundreds of molecular markers simultaneously throughout the genome. It can therefore be used to create genomic maps in plant breeding programs, especially in the context of studying traits with complex inheritance, and to analyze genetic diversity and expand information on the structure of the population of crops [20,21].

Genome wide association studies (GWAS) are a useful tool for the identification of candidate genes, especially when combined with QTL mapping to validate loci for quantitative traits. Zila et al. [22,23] conducted GWAS tests on maize to detect SNPs associated with increased resistance to fusarium. They identified ten SNP markers significantly associated with resistance to this pathogen [22,23]. Zila et al. [22] identified defense response SNPs in or around five genes that had not previously been correlated with disease resistance, but whose predicted gene functions involved a programmed cell death pathway.

Genomic selection (GS) by incorporating associations of SNPs detected with GWAS is a promising tool to improve fusarium resistance in maize [24].

Therefore, the aim of the research is to identify new markers of the SilicoDArT and SNP type, which can be used for the mass selection of varieties resistant to fusarium.

## 2. Results

### 2.1. Phenotyping

The establishment of a field experiment with 186 corn inbred lines in two localities, Kobierzyce and Smolice, allowed for the necessary observations of the degree of cobs infestation by the fusarium. Inbred lines derived from hybrid varieties available on the Polish market were used for this study. They are characterized mainly by Dent grain types. Hybrids, from which inbred lines were derived, belonged to BSSS and non-BSSS origin groups, mainly Iodent and Lancaster. The method used to assess cob fusariosis infection was: the “visually moldy kernels” method. Table 1 shows the mean values of the eight observations according to the BBCH scale in two localities. The degree of infection of maize plants (cobs) by fusarium was presented on a 9-point scale. According to the COBORU scale (Central Research Centre for Cultivar Testing): 9—resistant, 1—susceptible. Due to favorable weather conditions during the entire growing season, most of the analyzed lines were highly resistant to fusarium (9). The most susceptible to infection were the lines from Smolice: S124, whose resistance was 6.0 in the field in Smolice and 5.7 in the field in Kobierzyce, and S140, whose resistance was 7.0 in the field in Smolice and 6.7 in the field in Kobierzyce (Table 1).

In the first stage, the analysis of variance was made. Analysis was carried out in terms of observations concerning the degree of plant infestation by the fusarium. The analysis of variance indicated that the main effect of lines, as well as line × location interaction, were statistically significant in the degree of plant infestation by the fusarium. The differences in the degree of plant infestation by the fusarium between locations were not significant (Table 2). Significant correlations were also found between the degree of plant infection by the fusarium in Smolice and Kobierzyce (*r* = 0.8898, *p* < 0.001).

### 2.2. DNA Isolation

The efficiency of a single isolation using the Wizard^®^ (Madison, WI, USA) Genomic DNA Purification Kit was very good and ranged from 107 ng/µL for line 16 to 690 ng/µL for line 159. The purity of individual DNA samples allowed for their direct medium use for next-generation sequencing. The purity ranged from 1.7 to 2.0 for both 260/280 and 260/230 absorbance. Immediately before sending the sample, the concentration was adjusted to the same 100 µg/µL.

### 2.3. Genotyping

**Figure 1:**
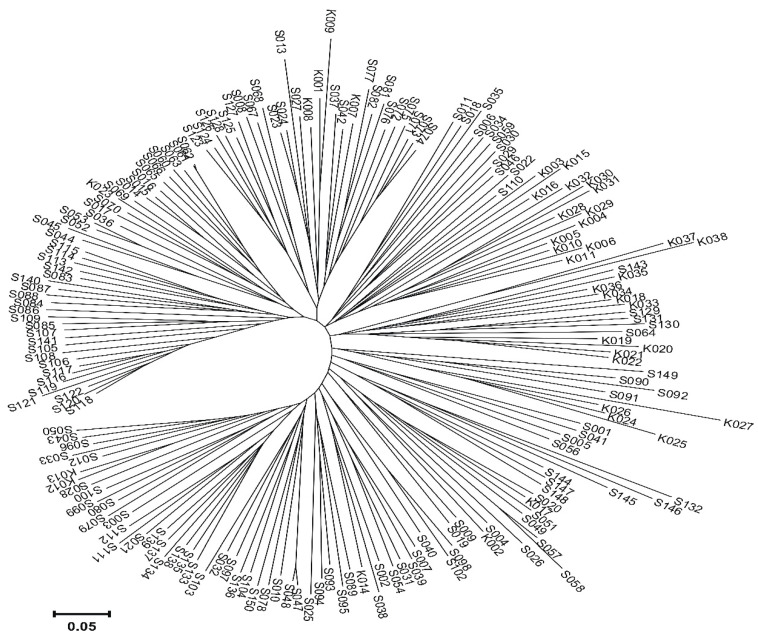
Dendrogram of genetic similarity between the analyzed lines, determined based on the identified molecular markers SilicoDArT and SNP.

Next-generation sequencing was made on 186 lines. The same lines were also observed eight times, under field conditions, for the infestation of corn cobs by fungi of the *Fusarium* genus. By performing sequencing analyzes, molecular markers SilicoDArT in the amount of 53,031 and SNP in the amount of 28,571 were identified. These markers were used to estimate the genetic similarity between the analyzed corn inbred lines (Figure 1). We can generally distinguish four main groups. The first group includes two lines from Plant Breeding (PB) in Kobierzyce (K037 and K038). These lines are 63% similar. The second group is also made up of two lines (S145 and S132), 51% similar to each other, coming from PB in Smolice (Figure 1). In the third large group, we can distinguish three subgroups. There are 25 lines in the first sub-group, 23 lines in the second, and 25 lines in the third. In all these subgroups lines derived from PB in Smolice constitute 87.5%, and the remaining 12.5% are lines derived from PB in Kobierzyce (Figure 1). The fourth main group is also made up of three subgroups (109 lines in total). Genotypes with PB in Kobierzyce (65%) dominate in the first subgroup, while in the second and third subgroups with PB in Smolice. When analyzing the dendrogram, it can be noticed that the lines are grouped depending on belonging to a given breeding company, moreover, the lines from PB in Smolice show greater similarity with each other than with the lines from Kobierzyce and vice versa, the lines from Kobierzyce are more similar. To each other than to the line from Smolice.

### 2.4. Associative Mapping Using GWAS Analysis

Of the 81,602 molecular markers (53,031 SilicoDArT and 28,571 SNPs) obtained by next-generation sequencing, 2962 (321 SilicoDArT and 2641 SNPs) are significantly related to the resistance of maize plants to ear rot were selected (Table 3). In order to narrow down the number of markers for physical mapping, seven were selected from all significant ones, which were significant at the level of 0.001. Analysis of variance indicated that the main effects of line, as well as location-by-line interaction, were significant for the degree of plant infestation by the fusarium.

### 2.5. Physical Mapping and Functional Analysis of Gene Sequences

From 2963 (321 SilicoDArT and 2641 SNP) markers significantly related to plant resistance in the fusarium, seven (five Silico DArT and two SNP) significant at the level of 0.001 were selected (Table 4). An attempt was also made to determine the location of the selected markers SilicoDArT and SNP. As a result of the analysis, it was found that two out of seven (15,097—DArT and 58,771—SNP) of the selected markers are located inside the genes, which are described in Table 4. In the case of the remaining markers, their location and distance from the closest genes were given. Marker 15,097 is anchored to the gene encoding the putrescine hydroxycinnamyltransferase protein, while marker 58,771 is anchored to the gene encoding the peroxidase precursor 72 (Table 4) (Figure S1).

### 2.6. Design of Primers for Identified SilicoDArT and SNP Polymorphisms Associated with Fusarium Resistance of Maize Plants

After determining the location of the seven selected DArT and SNP markers, an attempt was made to design primers that will be used for their identification. The designed primers are presented in Table 5. In the next year of research, the polymerase chain reaction (PCR) conditions will be refined in order to develop a methodology that will be used to identify the selected markers. In the following years, these markers can be used in breeding programs to select varieties resistant to fusarium.

## 3. Discussion

Ear rot is a fungal disease that occurs in many parts of the world and is considered to be one of the main factors affecting the size and quality of the obtained grain yield. It is caused by fungi belonging to the genus *Fusarium*, mostly *F. culmorum*, *F. graminearum*, and *F. verticillioides* [25]. *Fusarium* spp. infects maize grain, the aboveground parts of the entire plant become infected, leading to significant yield losses and deterioration in maize quality [26].

*Fusarium graminearum* invokes ear rot in maize, and was the main cause of maize cob fusariosis, among others, in Canada [27], China [28], and also in Europe in Italy [5,25].

Weather conditions are a factor that affects the infestation of cereal grains by fusarium to a greater extent than the differentiated farming systems. This theory is supported by studies by Champeil et al. [29]. Fusariosis risk assessment and models to predict its occurrence are based on weather conditions from flowering to early milk [30]. The weather conditions in the observation area in 2021 were not conducive to the spread of fungal diseases. June and July 2021 turned out to be dry (June 52.7 mm; July 65 mm) and warm (June 19.3 °C; July 20.9 °C). There were also no intense infestations of the European corn borer (*Ostrinia nubilalis*), which feed on maize, increasing its susceptibility to fusarium. Light traps and charts showing butterfly flight dynamics from previous years were used to estimate intense infestations of European corn borer. The increased infestation of maize by the fusarium was observed only in August, which was caused by a large amount of rainfall (140.1 mm) and a quite high temperature (17 °C). The very dry months of September (42.3 mm) and October (19.2 mm) inhibited the development of fungal diseases, including ear rot. Therefore, all analyzed lines were characterized by high resistance.

In this study, due to favorable weather conditions during the entire growing season, most of the analyzed lines were characterized by high resistance to fusarium (9), on a scale from 1-susceptible to 9-resistant. The most susceptible to infection were the lines from Smolice: line S124, the resistance of which was 6.0 in the experimental plots in Smolice and 5.7 in the experimental plots in Kobierzyce, and the line S140, the resistance of which was 7.0 in the plots in Smolice and 6.7 in the plots in Smolice in Kobierzyce.

Secondary metabolites of fungi of the genus *Fusarium* are highly harmful to humans and animals (especially pigs), causing disease and even death. *F. graminearum* produces deoxynivalenol—DON and zearalenone—ZEA, while *F. verticillioides* produces fumonisins—FUM.

Exposure of farm animals to the action of zearelenone leads to disorders of the genitourinary system, while acute or chronic poisoning can cause permanent damage to the organs of the reproductive system. Contamination of food of animal origin (mainly milk and meat) with mycotoxins from fusarium is currently low due to the constant monitoring of these products in terms of their safety [31]. Pigs and poultry are the most sensitive to mycotoxin contamination of feed [32]. Poultry is less sensitive to the fumonisin content of the feed than pigs and horses. This is related to the difference in the degree of absorption of this mycotoxin in the gastrointestinal tract [33]. Moreover, it has been noted that there is also a varied sensitivity to the fumonisin content among poultry. Turkeys and ducks are much more susceptible to poisoning than chickens [34]. Horses fed with fodder containing maize contaminated with aflatoxin died, and after the performed necrosis, extensive liver necrosis was found [33].

Therefore, one of the most recommended methods of plant protection against diseases, including fusarium, is resistance breeding [2]. There is a strong need to search for sources of resistance that could be used in further breeding works. It is also important to identify new resistance genes and their associated molecular markers.

New genes and molecular markers can be identified using Next Generation Sequencing (NGS). The most common NGS techniques include pyrosequencing 454 [35], the Solex technique (Ilumina), the SOLiD platform (Applied Biosystems), Polonator (Dover/Harvard), and the HeliScope Single Molecule Sequencer (Helicos). These technologies provide inexpensive whole genome sequence readings through the use of methods such as chromatin immunoprecipitation, mutation mapping, polymorphism detection, and detection of non-coding RNA sequences [36]. Sequencing methods such as RAD (Restriction site Associated DNA) [37], MSG (Multiplexed Shotgun Genotyping) [38], and BSRSEq (Bulked segregant RNA-Seq) [39] allow the identification of a large number of markers and allow for a more accurate study of many loci in a small number of samples. The method using the Ilumina approach gave rise to the development of GBS procedures [40] as well as DarTseq [41].

As a result of next-generation sequencing, the study authors identified 81,602 molecular markers (53,031 SilicoDArT and 28,571 SNP). From among them, they selected 2962 (321 SilicoDArT and 2641 SNP) significantly related to the resistance to ear rot. In order to narrow down the number of markers for physical mapping from the pool of all significant ones, they chose seven that were significant at the level of 0.001.

Marker Assisted Selection (MAS) allows for a reduction in financial outlays and an increase in productivity. By increasing the efficiency of selecting varieties for crossbreeding, breeders can improve breeding programs in a shorter time [42]. Salah et al. [43] identified the resistance/QTL genes on maize fusarium linked to the markers RAPD (OPA02), ISSR (AD8), SSR (SSR93, SSR105, SSR225, and SSR337), and STS (STS03) using MAS. The SSR and STS markers were shown to be on chromosome 10 [43]. The use of SNP markers linked to the features of the yield structure in maize and barley showed greater precision than methods based on the study of metabolic pathways [44]. A useful tool for identifying candidate genes and their associated molecular markers is genome wide association studies (GWAS). Zila et al. [23] conducted GWAS tests on maize to detect SNPs associated with increased resistance to fusarium. Zila et al. [23] identified 10 SNP markers significantly associated with resistance to this pathogen.

In this study, out of 2963 (321 SilicoDArT and 2641 SNP) markers significantly related to plant resistance in fusarium, seven (five Silico DArT and two SNP) significant at the level of 0.001 were selected. In order to identify the selected markers, primers were designed. As a result of the analysis, it was found that two out of seven selected markers (15,097—DArT and 58,771—SNP) are located inside genes. Three markers are situated inside the genes. Marker 553 is situated inside the fourth exon of the GDSL esterase/lipase At4g01130 precursor. The detailed analysis shows that the marker is located in the alanine codon (77A). This amino acid position has been not linked with any active sites of the enzyme. However, the detection of DArT markers does not inform about the type of mutation causing restriction site loss. Marker 15,097 is anchored in the *putrescine hydroxycinnamoyltransferase* gene (*LOC103649226*). The polymorphism is situated in upstream UTR and may have a potential influence on the promotor and the regulation of the expression of this gene. Marker 58,771 is anchored in the intron of two genes: *peroxidase precursor* gene *72* (*LOC100282124*) and pentatricopeptide repeat-containing protein At5g57250, mitochondrial (*LOC103649988*). In the case of *gene At5g57250*, the polymorphism may disrupt the AG motif at the end of the intron and may result in the blocking of the splicing process of this intron.

Polyamines, such as putrescine, spermidine, and spermine, are small basic molecules with two or more primary amino groups. Ubiquitous in nature, they are believed to be important growth regulators in both eukaryotic and prokaryotic cells [45]. In plants, in addition to free polyamines, polyamines are conjugated with hydroxycinnamic acids to produce acylated polyamines (polyamine conjugates), which are also referred to as hydroxy-cinnamic acid amides (HCAAs) [46]. So far, a number of acyltransferases responsible for amide formation with hydroxycinnamic acids have been detected in different plants, belonging to them as well as putrescine N-hydroxycinnamoyltransferase. This enzyme belongs to the family of transferases, specifically those acyltransferases transferring groups other than aminoacyl groups. Tanabe et al. [47] found in their research on rice that the enzyme putrescine N-hydroxycinnamoyltransferase is highly expressed in rice roots and flowers in response to the stress of pest attack. As early as 2006, Chen and others [48] wrote about the role of putrescine N-hydroxycinnamoyltransferase in tomato immune processes. Wang et al. [49], based on quantitative trait loci mapping and genome-wide association study, identified a single-nucleotide polymorphism locus highly associated with variation in the severity of Rp1-D21-induced HR-hypersensitivity response. From a previous two maize genes encoding hydroxycinnamoyltransferase (HCT; a key enzyme involved in lignin biosynthesis) homologs, termed HCT1806 and HCT4918, were adjacent to this single-nucleotide polymorphism.

The most important peroxidases are the cationic peroxidase from *Zinnia elegans* (ZePrx). This enzyme is responsible for the final stage of the plant’s lignification. Bibliographic evidence suggests that *Arabidopsis* 72 peroxidase (AtPrx72), which is a ZePrx homolog, may also play an important role in lignification [50]. Less than two years later, Fernández-Pérez et al. [51] stated that the *Arabidopsis* genome encodes for 73 peroxidases, among which AtPrx72 has been shown to participate in lignification. It is well known that lignin is a polymer composed of derivatives of phenolic alcohols. It is a substance that increases the density of wood cells and thus increases the stiffness of the cell wall, thanks to which it is resistant to mechanical factors and is a barrier to pathogens, including fungi of the *Fusarium* genus. Lanubile et al. [52] showed that in resistant maize seedlings, before infection, the expression of ascorbate peroxidase was higher than in susceptible seedlings, and the enzyme was activated after pathogen infection.

As indicated by the above results, markers (15,097—DArT and 58,771—SNP) of both genes can be used in breeding programs to select lines resistant to fusarium. The remaining five of the seven selected markers will be tested on susceptible and fusarium resistant maize lines to also determine their suitability for the selection of resistant genotypes.

## 4. Materials and Methods

### 4.1. Plant Material

The plant material consisted of 186 inbred maize lines. The lines came from experimental plots belonging to two Polish breeding companies: Plant Breeding Smolice, Smolice, Ltd., Co., Poland Plant Breeding and Acclimatization Institute—National Research Institute Group (51°41′23.16″ N, 17°4′18.241″ E) and Małopolska Plant Breeding Kobierzyce, Kobierzyce, Ltd., Co., Poland (50°58′19.411″ N, 16°55′47.323″ E).

### 4.2. Methods

#### 4.2.1. Phenotyping

A field experiment with 186 corn inbred lines was established in two localities of Kobierzyce and Smolice. The plant material was sown on 10 m^2^ of experimental plots, in the system of complete randomly selected blocks, in three replications. In the course of the experiments, observations were made concerning the degree of infection of maize cobs by the fusarium. The observations were carried out on eight dates: term 1—development of the first blister stage kernels, which contain about 16% of dry matter (BBCH 71), date 2—the beginning of early milk (BBCH 73), term 3—milk stage; middle kernels milky, contain about 40% of dry matter (BBCH 75), term 4—nearly all kernels have reaches final volume (BBCH 79), date 5—the beginning of the kernel’s denting maturity, kernels soft; 45% of dry matter (BBCH 83), date 6—full denting maturity of the kernels, kernels with a typical color, they contain about 55% of dry matter (BBCH 85), term 7—physiological maturity, visible black layering at the base of the kernel contain about 60% of dry matter (BBCH 87), date 8—full maturity, hard and shiny kernels contain about 65% dry weight (BBCH 89).

The meteorological conditions in the growing season of 2021 were favorable for the growth and development of maize, although the frosts in April delayed sowing. May, which is very important for the growth and development of maize, should be classified as cool (12 °C) and humid because the amount of rainfall was 76 mm. Contrary to May, June, and July 2021. turned out to be dry (June 52.7 mm; July 65 mm) and warm (June 19.3 °C; July 20.9 °C). Dry and warm weather was not conducive to the spread of fungal diseases during this period. Intensive infestation of European corn borer (*Ostrinia nubilalis*), was also not observed. European corn borer feeds on maize and increases its susceptibility to fusarium by laying eggs from mid-June to the end of August. In August, an increased infestation of maize by fusarium was observed, which was caused by a large amount of rainfall (140.1 mm) and a quite high temperature (17 °C). The very dry months of September (42.3 mm) and October (19.2 mm) inhibited the development of fungal diseases, including ear rot. Therefore, all analyzed lines were characterized by high resistance.

#### 4.2.2. DNA Isolation

Wizard^®^ Genomic DNA Purification Kit from Promega was used to isolate DNA from 186 inbred lines. Tissue from 7-day-old seed leaves was used for DNA extraction. Immediately after isolation, the concentration and purity of the isolated DNA samples were determined using the DeNovix DS-11 spectrophotometer. In the next step, the template DNA was diluted with distilled water to 100 ng/µL and stored at −80 °C until sequencing.

#### 4.2.3. Genotyping

The methodology used for next-generation sequencing was described in detail in the publication presenting the research by Tomkowiak et al. [53]. The DArTseq analysis was performed at Diversity Arrays Technology Pty Ltd. (Canberra, Australia). DNA sample digestion/ligation reactions were processed according to Kilian et al. [54] but replacing a single PstI-compatible adaptor with two adaptors corresponding to PstI- and NspI-compatible sequences and moving the assay on the sequencing platform as described by Sansaloni et al. [40]. The PstI-compatible adapter was designed to include Illumina flow cell attachment sequence, sequencing primer sequence, and “staggered” varying length barcode region, similar to the sequence reported by Elshire et al. [41]. The reverse adapter contained a flowcell attachment region and NspI-compatible overhang sequence. Only “mixed fragments” (PstI–NspI) were amplified in PCR using the following reaction conditions: Denaturation 1 min at 94 °C, followed by 30 cycles of 94 °C for 20 s, 57 °C for 30 s, and 72 °C for 45 s, and the final elongation 72 °C for 7 min. After PCR equimolar amounts of amplification products from each sample of the 96-well microtiter plate are bulked and applied to c-Bot (Illumina, San Diego, California, United States) bridge PCR followed by sequencing on Illumina Hiseq2500. The sequencing (single read) was run for 78 cycles. Sequences generated from each lane were processed using proprietary DArT analytical pipelines. In the primary pipeline, the fastq files were first processed to filter away poor quality sequences, applying more stringent selection criteria to the barcode region compared to the rest of the sequence. In that way, the assignments of the sequences to specific samples carried in the “barcode split” step were very reliable. Approximately 2,500,000 (±7%) sequences per barcode/sample were used in marker calling. Finally, identical sequences were collapsed into “fastqcall files”. These files were used in the secondary pipeline for DArT PL’s proprietary SNP and SilicoDArT (presence/absence of restriction fragments in representation) calling algorithms (DarTsoft14). For the association analysis, only DarT sequences meeting the following criteria were selected: One SilicoDArT and SNP within a given sequence (69 nt), minor allele frequency (MAF) >0.25, and the missing observation fractions <10%.

#### 4.2.4. Statistical Analysis

The normality of the distribution of the degree of infection of the maize line by the fusarium was tested using Shapiro–Wilk’s normality test to check whether the analysis of variance (ANOVA) met the assumption that the ANOVA model residuals followed a normal distribution. The homogeneity of variance was tested using Bartlett’s test. A two-way analysis of variance (ANOVA) was carried out to determine the main effects of line, location, and line×location interaction on the variability of the degree of infection of the maize line by the fusarium. The genetic similarity for each pair of the investigated lines was estimated based on the coefficient proposed by Nei and Li [55]. The lines were grouped hierarchically using the unweighted pair group method of arithmetic means (UPGMA) based on the calculated coefficients [56]. The relationships between the lines were presented in the form of a dendrogram [57,58]. The relationship between the degree of infection of the maize line by the fusarium in both locations was assessed based on Pearson’s correlation coefficients and tested with the *t*-test [59]. All analyses were conducted in Genstat 18.2 (VSN International Ltd., Hemel Hempstead, UK).

#### 4.2.5. Associative Mapping Using GWAS Analysis

Associative mapping was performed using GWAS analyzes. An attempt was made to link the observations of the degree of ear rot infestation by the fusarium, 186 inbred maize lines with the molecular markers SNP and SilicoDArT obtained from the DArTseq analysis. On the basis of the GWAS analysis, the silicoDArT and SNP markers were selected for further studies that showed the highest level of significance, i.e., those that were most strongly associated with the resistance of maize to ear rot. The lower limit of the selected significance level results from the Manhattan plot charts, which graphically present the results of the association studies.

#### 4.2.6. Physical Mapping

The databases were searched to find sequences with high homology to the selected sequences of the silicoDArT and SNP markers. The bioinformatics tool BLAST (Basic Local Alignment Search Tool) was used for this purpose. BLAST is one of the most frequently used programs of this type due to the use of a heuristic mechanism, it does not compare the entire sequence, but the shorter fragments of both sequences. The analyzes were performed using URGI (Unité de Recherche Génomique Info) with a completely sequenced maize genome. The genome was sequenced from inbred lines from hybrid varieties available on the Polish market. The location on the chromosome of the searched sequences, which were similar to the analyzed sequences, was indicated, and their physical location was determined. To identify a region containing sequences similar to the analyzed sequences, an overall probability was calculated from the e-value (e-value) of each chromosome. The sequences of all genes in the designated area on the chromosome were further analyzed.

#### 4.2.7. Functional Analysis of Gene Sequences

In order to obtain information about the biological function of genes located in the designated area of chromosomes, a functional analysis was performed using the Blast2GO program. The sequences of all genes located in the area of chromosomes determined on the basis of the BLAST analysis performed on the URGI website were analyzed.

#### 4.2.8. Designing Primers for Identified SilicoDArT and SNP Polymorphisms Related to Fusarium Resistance

The Primer 3 Plus program was used to design the starters.

## 5. Conclusions

The introduction of next-generation sequencing (NGS) methods was a great breakthrough that revolutionized the world of molecular biology techniques. It is difficult to say how quickly NGS will become a standard in breeding programs, but it is known for certain that it has a wide range of applications. Currently, this technique is used for genomes and transcriptomes sequencing, studying protein-DNA/RNA interactions, checking the degree of methylation, as well as for metagenomic studies. In this study, the next-generation sequencing resulted in obtaining the molecular markers SilicoDArT (53,031) and SNP (28,571), on the basis of which the genetic distance between the analyzed lines was estimated. When analyzing the dendrogram, it can be noticed that the lines from PB Smolice show greater similarity with each other than with the lines from PB Kobierzyce, and conversely, the lines from Kobierzyce are more similar to each other than with the lines from Smolice. In total, 81,602 molecular markers were obtained, of which, as a result of the associative mapping, 2962 (321 SilicoDArT and 2641 SNP) significantly related to plant resistance to fusarium were selected. Out of 2962 markers significantly related to plant resistance in the fusarium, seven markers (SilicoDArT, SNP) were selected, which were significant at the level of 0.001. These markers were used for physical mapping. As a result of the analysis, it was found that two out of seven selected markers (15,097—SilicoDArT and 58,771—SNP) are located inside genes. These markers are located on chromosomes 2 and 3, respectively. Marker 15,097 is anchored in the gene encoding putrescine N-hydroxycinnamoyltransferase, while marker 58,771 is anchored in the gene encoding peroxidase 72 precursor. These genes may be related to plant resistance to fusarium. Therefore, the markers 15,097 (SilicoDArT) and 58,771 (SNP) can be used in breeding programs to select lines resistant to fusarium.

## Figures and Tables

**Table 1:** The degree of infection of the maize line by the fusarium in Kobierzyce and Smolice (average of all observations made).

Line Number	Line Name	The Degree of Infection (9-Point Scale)		Line Number	Line Name	The Degree of Infection (9-Point Scale)		Line Number	Line Name	The Degree of Infection (9-Point Scale)	
		Smolice	Kobierzyce			Smolice	Kobierzyce			Smolice	Kobierzyce
1	S001	7	7.7	63	S063	9	9	125	S125	9	8.7
2	S002	8	8.3	64	S064	9	9	126	S126	9	8.7
3	S003	9	9	65	S065	7	8.7	127	S127	9	9
4	S004	9	9	66	S066	9	9	128	S128	7	7.3
5	S005	9	9	67	S067	9	9	129	S129	8	8.3
6	S006	9	9	68	S068	9	9	130	S130	9	8.7
7	S007	9	8.7	69	S069	9	9	131	S131	8	8.7
8	S008	8	8	70	S070	9	9	132	S132	7	7
9	S009	9	9	71	S071	9	9	133	S133	8	8.3
10	S010	9	9	72	S072	9	9	134	S134	8	7.5
11	S011	9	9	73	S073	9	8.7	135	S135	9	8.7
12	S012	9	9	74	S074	9	8.7	136	S136	8	9
13	S013	7	7.7	75	S075	9	9	137	S137	8	8.3
14	S014	9	9	76	S076	9	8.7	138	S138	9	9
15	S015	9	9	77	S077	9	8.7	139	S139	9	8.7
16	S016	8	8.3	78	S078	9	9	140	S140	7	6.7
17	S017	9	9	79	S079	9	9	141	S141	9	9
17	S018	8	9	80	S080	9	9	142	S142	9	9
18	S019	9	9	81	S081	9	9	143	S143	9	9
20	S020	9	8.7	82	S082	9	9	144	S144	9	8.7
21	S021	9	9	83	S083	9	9	145	S145	9	9
22	S022	8	8.3	84	S084	9	9	146	S146	9	9
23	S023	9	9	85	S085	9	8.7	147	S147	9	9
24	S024	8	9	86	S086	8	9	148	S148	9	8.7
25	S025	9	9	87	S087	8	9	149	S149	8	8
26	S026	9	9	88	S088	9	9	150	S150	9	9
27	S027	9	9	89	S089	9	9	151	K001	9	9
28	S028	9	9	90	S090	9	9	152	K002	9	8.7
29	S029	9	9	91	S091	8	8.3	153	K003	9	8.7
30	S030	9	9	92	S092	9	9	154	K004	9	9
31	S031	9	8.7	93	S093	8	8.7	155	K005	8	8.3
32	S032	9	9	94	S094	9	8.7	156	K006	8	8
33	S033	9	9	95	S095	9	8.7	157	K007	9	8.7
34	S034	9	8.7	96	S096	9	9	158	K008	9	9
35	S035	7	8.3	97	S097	9	9	159	K009	8	8.3
36	S036	9	9	98	S098	9	9	160	K010	8	8.7
37	S037	9	8.7	99	S099	9	9	161	K011	9	9
38	S038	9	9	100	S100	9	8.7	162	K012	9	9
39	S039	8	9	101	S101	9	9	163	K013	9	9
40	S040	9	9	102	S102	9	9	164	K014	9	9
41	S041	9	9	103	S103	9	9	165	K015	9	9
42	S042	9	9	104	S104	9	9	166	K016	8	8.3
43	S043	9	9	105	S105	9	8.7	167	K017	9	8.7
44	S044	9	9	106	S106	8	8.3	168	K018	9	9
45	S045	9	8.7	107	S107	8	7.7	169	K019	9	9
46	S046	9	9	108	S108	9	8.7	170	K020	8	8
47	S047	9	9	109	S109	9	9	171	K021	9	9
48	S048	8	9	110	S110	9	8.7	172	K022	8	8.3
49	S049	7	7	111	S111	8	8	173	K023	9	9
50	S050	9	9	112	S112	9	8.7	174	K024	9	9
51	S051	7	7.3	113	S113	9	8.7	175	K025	9	9
52	S052	9	9	114	S114	9	8.7	176	K026	9	9
53	S053	8	8.3	115	S115	9	9	177	K027	9	9
54	S054	9	9	116	S116	9	9	178	K028	7	7.7
55	S055	9	9	117	S117	9	9	179	K029	9	9
56	S056	9	9	118	S118	9	9	180	K030	8	8.3
57	S057	9	9	119	S119	9	9	181	K031	9	9
58	S058	9	8.7	120	S120	8	8	182	K032	8	8.3
59	S059	9	9	121	S121	9	9	183	K033	9	9
60	S060	9	9	122	S122	9	8.7	184	K034	9	9
61	S061	9	8.7	123	S123	8	8.3	185	K035	9	9
62	S062	9	9	124	S124	6	5.7	186	K036	9	9

**Table 2:** Values of *F*-statistics from two-way analysis of variance for the degree of plant infestation by the fusarium.

Source of Variation	The Number of Degrees of Freedom	*F* Statistic
Location	1	0.18
Lines	251	16.22 ***
Location × line interaction	251	25.73 ***

*** *p* < 0.001.

**Table 3:** Molecular markers of SilicoDArT and SNP significantly related to the resistance of maize to ear rot (significant associations selected at *p* < 0.05 corrected for multiple testing by the Benjamini-Hochberg method).

Location		Kobierzyce	Smolice	Total
The number of significant markers	SilicoDArT	136	185	321
	SNP	1067	1574	2641
	Total (Silico DArT and SNP)	1203	1759	2962
Minimal effect	SilicoDArT	−1.234	−0.279	
	SNP	−1.469	−0.305	
	Total (Silico DArT and SNP)	−1.469	−0.305	
Maximal effect	Silico DArT	1.381	0.269	
	SNP	1.574	0.311	
	Total (Silico DArT and SNP)	1.574	0.311	
Average effect	Sicico DArT	0.092	0.039	
	SNP	−0.043	0.008	
	Total (Silico DArT and SNP)	−0.028	0.011	
Total effect	Silico DArT	12.483	7.146	
	SNP	−46.064	12.99	
	Total (Silico DArT and SNP)	−33.581	20.136	

**Table 4:** Characteristics and location of markers significantly related to plant resistance to fusarium.

Marker	Marker Type	Chromosome	Marker Location	Candidate Genes
553	DArT	Chr9	19345104	A marker that is anchored in the gene *GDSL esterase/lipase At4g01130* precursor uncharacterized precursor of the protein) (LOC100273960)
10382	DArT	Chr10	149495362	1182 bp at 5′ side: ubiquitin carboxyl-terminal hydrolase 3 (LOC100191221) 1718 bp at 3′ side: Heavy metal transport/detoxification superfamily protein (LOC100501931)
13242	DArT	Chr1	292840905 292841155 292841283	Within the tRNA Cys, 66,700 bp at 5′ side: fasciclin-like arabinogalactan protein 16 precursor (LOC100191430) 90,541 bp at 3′ side: calcium dependent protein kinase 11 (LOC103644148)
15097	DArT	Chr2	203171066	A marker that is anchored in *putrescine hydroxycinnamyltransferase* gene (LOC103649226)
15156	DArT	Chr5	215026162	50,422 bp at 5′ side: photosynthetic NDH subunit of subcomplex B 4 chloroplastic (Loc100276619) 408 bp at 3′ side: pseudogene (LOC103627720) and 40,871 bp: expansin alpha precursor 2 (LOC542648)
58153	SNP	Chr9	145274999	1499 bp at 5′ side: histon h2a (LOC103639303) 2328 bp at 3′ side: histon h2b.1-similar (LOC103639303)
58771	SNP	Chr3	40548812	A marker that is anchored the peroxidase precursor gene 72 (LOC100282124) and pentatricopeptide repeat-containing protein At5g57250, mitochondrial (LOC103649988)

**Table 5:** Sequences of the designed primers for the identification of newly selected markers significantly related to the analyzed features.

Marker	Primer Sequences		Annual Temperature (°C)	Product Size (bp)
	Forward	Reverse		
553	TTGTCGACGTACACGACCG	TTCGGGTGCGTGAAAAGCTA	60	116
10,382	GCAGTGCGTCGTGCAGT	AAGCCGATCGAGTTTGTGTTT	58	91
13,242	ACCTGCAGATCAATAGTCAC	GGACCCTTTGTATCGAAAA	52	122
15,097	GGCTCACCTTCCCGTTCTAC	GTACGAAGGCACCAGGAACA	59	107
15,156	CCGACATCAAATGTCACAGCA	TGAGAAGACGACGACGAAGC	59	151
58,153	ACTGCAGTATGGGACCACAA	TGAAACATGCACCAAAATAAAATCC	57	100
58,771	TGCTAGCACAAGTGCATTTCAA	TGAAGGTGTTGCAAGCGAAT	58	103

## Data Availability

The datasets generated during and/or analyzed during the current study are available from the corresponding author on reasonable request.

## Supplementary Materials

The following supporting information can be downloaded at: https://www.mdpi.com/article/10.3390/ijms23116105/s1.
