# Supplementary material for: Associative and Physical Mapping of Markers Related to Fusarium in Maize Resistance, Obtained by Next-Generation Sequencing (NGS)
Source: Int J Mol Sci. 2022 May 29;23(11):6105. doi: 10.3390/ijms23116105 (PMC9181084; doi:10.3390/ijms23116105)
Supplement: Supplementary file 1 [file ijms-1703303-supplementary.pdf › ijms-1703303-supplementary.pdf]

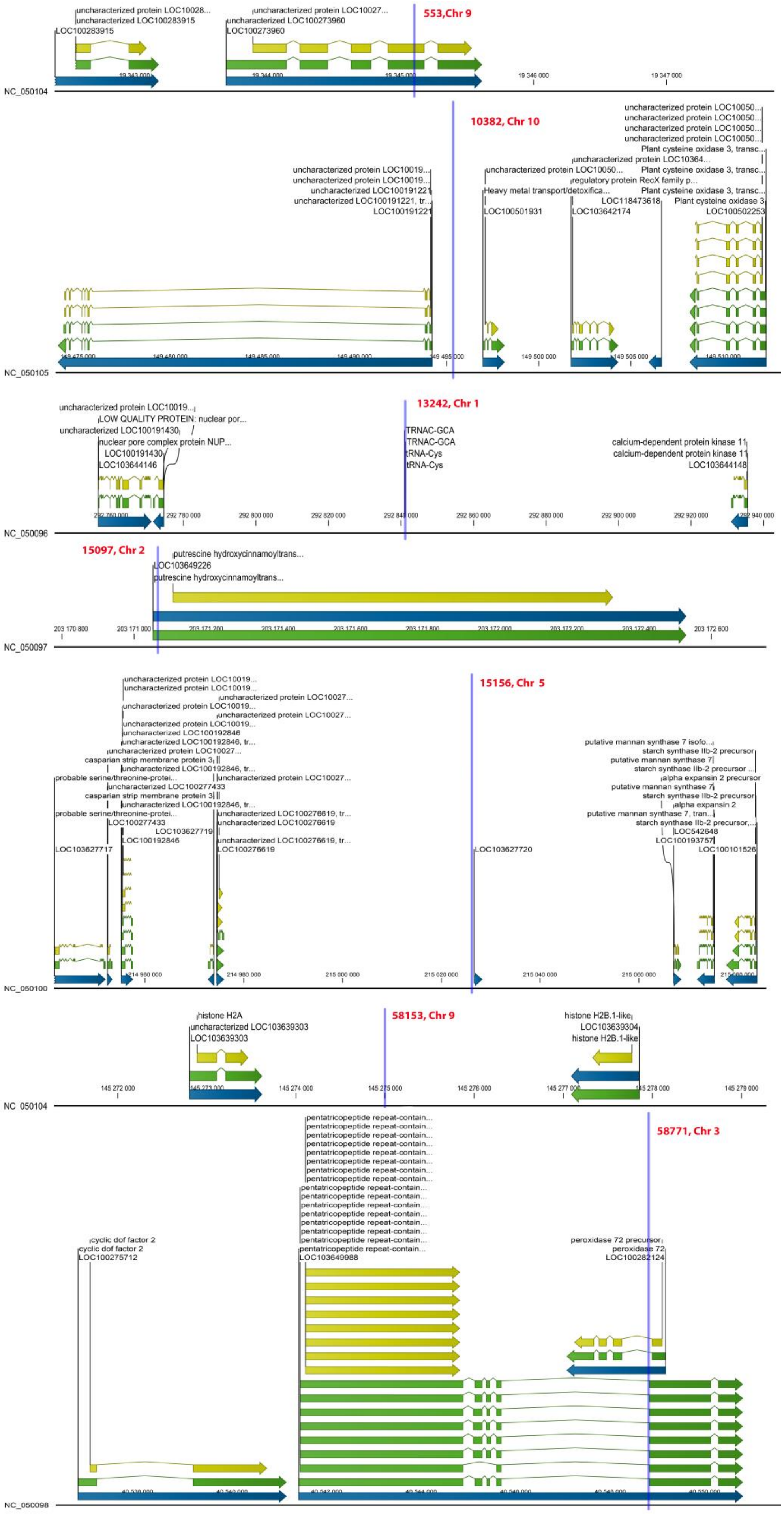

Figure S1. Markers on chromosomes with adjacent genes.
